# Predictors of poor quality of life for patients discharged from acute psychiatric care in Alberta

**DOI:** 10.3389/fpsyt.2025.1619292

**Published:** 2025-08-13

**Authors:** Ernest Owusu, Wanying Mao, Reham Shalaby, Hossam Eldin Elgendy, Belinda Agyapong, Ejemai Eboreime, Mobolaji A. Lawal, Nnamdi Nkire, Peter H. Silverstone, Pierre Chue, Xin-Min Li, Wesley Vuong, Arto Ohinmaa, Valerie Taylor, Carla T. Hilario, Andrew J. Greenshaw, Vincent I. O. Agyapong

**Affiliations:** ^1^ Department of Psychiatry, University of Alberta, Edmonton, AB, Canada; ^2^ Department of Psychiatry, Dalhousie University, Halifax, NS, Canada; ^3^ Connect Care Clinical Operations Informatics Office, Alberta Health Services, Edmonton, AB, Canada; ^4^ School of Public Health, University of Alberta, Edmonton, AB, Canada; ^5^ Department of Psychiatry, Cumming School of Medicine, University of Calgary, Calgary, AB, Canada; ^6^ School of Nursing, University of British Columbia, Okanagan, BC, Canada

**Keywords:** anxiety, depression, mobility, pain and discomfort, quality of life

## Abstract

**Background:**

Quality of life (Qol) is a multi-dimensional concept composed of various dimensions, including mental and/or psychological well-being and physical and/or biological health. Objective: The objective of this study is to assess the prevalence and predictors of poor Qol outcomes across the five dimensions of the EQ-5D-5L, namely, mobility, self-care, usual activities, pain/discomfort, and depression/anxiety for individuals discharged from acute psychiatric care in Alberta.

**Methods:**

Multiple binary logistic regression models were performed to examine the association between sociodemographic variables and EQ-5D-5L dimensions.

**Results:**

Out of the 1106 participants, the majority were Caucasian, 61.6%, 25 years or less, 36.4%, females 54.8%, and had a high school diploma, 51.4%. The prevalence of depression/anxiety in the cohort is 89.2%. Caucasians were two times more likely to present with problems regarding pain/discomfort (OR=2.14; 95% C.I. 1.39 - 3.27) compared to Black participants. Also, retired participants were three times more likely to present with pain/discomfort (OR 3.18; 95% C.I. = 1.45 - 6.96) than those employed. Finally, participants with likely anxiety were almost two times more likely to present problems relating to self-care (OR=1.99; 95% C.I. = 1.41 - 2.81) compared with those who had unlikely anxiety.

**Conclusion:**

This study’s results highlight the complex interplay of demographic, socioeconomic, and mental health factors that influence various health-related problems. These findings underscore the importance of targeted, holistic health interventions that address physical and mental health needs.

## 1 Introduction

Quality of life (Qol) is a multidimensional concept composed of various dimensions, such as mental and/or psychological well-being, physical and/or biological health, social interactions, and environmental factors, such as nurturing and parenting style (1–3). In recent years, an increasing focus has been placed on examining how physical and mental health conditions affect overall Qol. The overall level of Qol is impacted by a variety of different factors, including both physical and mental health factors (4).

Globally, mental health disorders account for a significant amount of the public health burden caused by diseases (5, 6), and they significantly impact QoL (6, 7), leading to poorer health outcomes when compared to the general population (2, 3). In turn, QoL is affected by healthcare outcomes in psychiatric illness (8–10). Nonetheless, the direct contribution of mental health disorders to overall Qol remains uncertain, and a significant portion of the population continues to experience poor Qol despite the increased availability of treatments for mental health disorders (11). Deteriorated Qol, reduced capacity to work, and impoverished everyday life with few meaningful activities are most often the adverse outcomes of mental illness (12–15). There seems to be differences in Qol between individuals with different mental health challenges, with those with issues relating to depression having a profound effect on quality of life (Qol), disrupting multiple areas of daily functioning, such as physical health, social relationships, and general well-being (16). For these reasons, it is essential to better understand the relationship between low Qol and mental health disorders.

Several factors have been implicated in predicting low Qol among patients with mental health problems. These factors can be broadly categorized into personal-level factors, such as comorbidity, medical conditions, symptom severity, level of cognitive functioning, and social and environmental factors, including availability of social support, stigma and discrimination, socioeconomic status, and access to healthcare services. It has been suggested that stigma and discrimination can cause additional burdens and psychological difficulties, including lack of confidence, low self-esteem (17), more limited social networks (18), and reduced Qol (19). Additionally, the progression from mental health inpatient care to community living poses significant challenges for persons with mental health challenges (20–22), and consequently, some experience worsening symptoms relating to their mental health problems just before discharge (23). These factors may vary widely. For example, individuals with depression and anxiety report more significant impairment in functioning (24). The negative impacts of pain are also likely to impact Qol, regardless of the type or source of the pain (25), and which affect multiple areas including physical well-being (especially lack of energy and fatigue, sexual activity and sleep problems), psychological well-being (mainly decrease in positive emotions, cognitive activities and self-esteem), independence level (especially for mobility, activities of daily living), and environmental factors (especially physical security, availability of social services and job satisfaction) (26, 27). Additionally, Qol also varies by gender (28, 29), age (30–32), and cultural influences (33, 34). Despite this, there has been relatively little work in those admitted to acute psychiatric units, who often have more severe forms of mental health disorders. One study did examine this, but it primarily focused on the potential impact of seclusion or restraint during inpatient care. However, no difference was found in Qol before discharge (35). While some specific nursing interventions do appear to help this population after they are admitted (36, 37), they have not previously been linked to particular domains of Qol in psychiatric inpatients. Understanding this may help in the design of even more effective interventions. Thus, further identifying the factors contributing to these reduced Qol measures is essential to help design and implement appropriate interventions and supports, as the contributions to Qol in this population remain uncertain (38, 39).

In the present study, the prevalence of Qol was measured across the five dimensions of the EQ-5D-5L, namely mobility, self-care, usual activities, pain/discomfort, and depression/anxiety, for individuals being discharged from acute psychiatric care in Alberta. The study examined how these relate to a variety of demographic and diagnostic factors.

## 2 Methodology

### 2.1 Study setting and design

This study was conducted in the Canadian province of Alberta, which has a population of 4.7 million as of July 1, 2023 (40). The research occurred across the acute mental health units in three main cities: Edmonton, Calgary, and Grande Prairie. The data for this study were collected as part of an ongoing pragmatic stepped-wedge cluster-randomized, longitudinal trial (41) which aimed to assess the impact of supportive text messages (Text4Support) and peer support services (PSS) on individuals with mental illness following their discharge from acute mental health facilities in Alberta (41). Participants were recruited from the nine main sites, and this study analyzed the baseline datasets retrieved from the randomized trial.

### 2.2 Sample size calculation

We estimated using an online app, with a margin of error of +3%, an estimated inpatient population of 28571, and a confidence interval of 95% (42)., that the sample size needed to assess the prevalence of likely problems with mobility, self-care, usual activities, pain/discomfort and depression/anxiety would be 829.

### 2.3 Data collection

The data for this study were collected using RedCap (43)., an online platform for data collection. Participants were eligible if they were 18 or older, diagnosed with any mental health condition, ready for discharge from an inpatient mental health unit, owned a mobile device, could read English text messages, and could provide informed written consent.

The sociodemographic data collected included age, gender, ethnicity, education level, relationship status, and employment status. Also, clinical variables such as levels of depression and anxiety were collected at discharge using the PHQ9 and GAD-7, respectively. When using the PHQ-9 screening tool, a score of ten or higher indicating moderate, moderately severe and severe is recommended as a cut-off for identifying patients with likely MDD (44, 45). With the GAD-7, the scale has a range of scores from 0 to 21, with a score of ten or higher indicating moderate, moderately severe and severe is recommended as a cut-off for identifying patients with likely GAD (46). For this paper, GAD-7 scores between 10 and 21 were classified as ‘Likely Anxiety,’ and scores less than 10 as ‘Unlikely Anxiety.’ Also, PHQ-9 scores between 10 and 27 were classified as ‘Likely Depression’ and scores less than 10 were classified as ‘Unlikely Depression’. Data for this paper includes patients recruited between March 1, 2022, and November 5, 2023. After signing a paper-based consent form, data were collected from respondents before their discharge from the acute care units. All participants completed an online baseline survey with technical assistance from the research team members if needed. Phone numbers and healthcare numbers were used as primary identifiers to track responses across follow-up time points, with phone numbers specifically linking responses at the designated follow-up time points (3).

### 2.4 Outcome measures

This study’s primary outcome of interest was five components of the EQ-5D-5L scale (47, 48). The EQ-5D-5L is a widely used generic, preference-based measure of health-related quality of life (HRQL). It consists of two main components: a descriptive system and a visual analogue scale (EQ-VAS). The descriptive system evaluates five dimensions of health: mobility, self-care, usual activities, pain/discomfort, and depression/anxiety (48, 49). Each dimension in the EQ-5D-5L has 5 levels where 1 is no problem, 2 slight problems, 3 moderate problems, 4 severe problems, and 5 is extreme problems. For this paper, we compiled the responses into two main categories: “No Problems” (level 1) and “Problems” (levels 2-5). The scale is considered a reliable and valid instrument that can describe health status, which in many cases can apply to a broad range of populations with between 0.65 and 0.91 test-retest reliability (47, 50).

### 2.5 Statistical analysis

Data analysis for this study was performed using SPSS for Mac, version 25 (IBM et al., USA) (51). Descriptive statistics focused on the respondents’ sociodemographic and clinical characteristics against ethnicity status. Univariate analysis using a chi-square test was applied to determine the association between demographic and clinical factors and the five dimensions of the EQ-5D-5L instrument. Multiple binary logistic regression models were performed to examine all the variables’ association with likely problems with mobility, self-care, usual activities, pain/discomfort, and depression/anxiety among respondents. Variables with significant (p ≤ 0.05) or near significant (0.1 > p > 0.05) association on univariate analysis were included in the binary logistic regression models. Also, a correlational analysis was used to rule out any strong intercorrelations (Spearman’s correlation coefficient of 0.7 to 1.0 or −0.7 to −1.0) between the variables. The logistic regression model was employed to identify the significant predictors of having problems in each of the five dimensions. Confidence intervals and odds ratios (OR) were reported.

## 3 Results

1106 participants completed the baseline data and were included in this study.

**Table 1** depicts the distribution of participants’ sociodemographic and clinical characteristics against the ethnicity distribution. Most of the participants were Caucasian (61.6%), followed by Asian (11.3%), Black (10.8%), and the Indigenous population (9.2%), and Other constituting (7.1%). Out of the 1106 participants, the majority were 25 years or less (36.4%), female (54.8%), had a high school diploma (51.4%), single (59.0%), with those unemployed being (52.9%), and lived with family and friends (41.5%). The majority had a likely depression of (57.0%) and unlikely anxiety of (63.4).

**Table 1:** Demographic and clinical information on the participants.

Variables N (%)	Caucasians N= 681	Indigenous N= 102	Black N= 120	Asian N= 125	Other N= 78	Total N= 1106
Age categories (years)						
≤25 years 26–40 years **>**40 years	210 (30.8) 235 (34.5) 236 (34.7)	43 (42.2) 31 (30.4) 28 (27.5)	63 (52.5) 41 (34.2) 16 (13.3)	60 (48.0) 40 (32.0) 25 (20.0)	27 (34.6) 31 (39.7) 20 (25.6)	403 (36.4) 378 (34.2) 325 (29.4)
Gender						
Male Female Other Gender	285 (41.9) 376 (55.2) 20 (2.9)	33 (32.4) 65 (63.7) 4 (3.9)	54 (45.0) 64 (53.3) 2 (1.7)	54 (43.2) 68 (54.4) 3 (2.4)	42 (43.2) 33 (42.3) 3 (3.8)	468 (42.3) 606 (54.8) 32 (2.9)
Education categories						
Less than High School High School Diploma Post-Secondary Education Other	27 (4.0) 328 (48.2) 305 (44.8) 21 (3.1)	3 (2.9) 69 (67.6) 26 (25.5) 4 (3.9)	4 (3.3) 67 (55.8) 43 (35.8) 6 (5.0)	3 (2.4) 64 (51.2) 56 (44.8) 2 (1.6)	4 (5.1) 40 (51.3) 33 (42.3) 1 (1.3)	41 (3.7) 568 (51.4) 463 (41.9) 34 (3.1)
Current relationship status						
Single Separated/Divorced Partnered/Married/Common law. Widowed Other	366 (53.7) 66 (9.7) 220 (32.3) 8 (1.2) 21 (3.1)	69 (67.6) 4 (3.9) 25 (24.5) 1 (1.0) 3 (2.9)	91 (75.8) 4 (3.3) 20 (16.7) 1 (0.8) 4 (3.3)	80 (64.0) 6 (4.8) 37 (29.6) 1 (0.8) 1 (0.8)	46 (59.0) 7 (9.0) 21 (26.9) 1 (1.3) 3 (3.8)	652 (59.0) 87 (7.9) 323 (29.2) 12 (1.2) 32 (2.9)
Current employment status						
Student Employed Unemployed Retired Other	41 (6.0) 215 (31.6) 347 (51.0) 55 (8.1) 23 (3.4)	4 (3.9) 20 (19.6) 75 (73.5) 0 (0.0) 3 (2.9)	9 (7.5) 29 (24.2) 75 (62.5) 4 (3.3) 3 (2.5)	28 (22.4) 32 (41.0) 58 (46.4) 4 (3.2) 3 (2.4)	7 (9.0) 32 (41.0) 30 (38.5) 5 (6.4) 4 (5.1)	89 (8.0) 328 (29.7) 585 (52.9) 68 (6.1) 36 (3.3)
Current housing status						
Own Home Rented Accommodation Live with Family/Friends Couch/Shelter/Street/Other **Total**	173 (25.4) 233 (34.3) 236 (34.7) 38 (5.6)	10 (9.8) 43 (42.2) 42 (41.2) 7 (6.9)	8 (6.7) 26 (21.7) 75 (62.5) 11 (9.2)	20 (16.0) 20 (16.0) 78 (62.4) 7 (5.6)	9 (11.5) 36 (46.2) 28 (35.9) 5 (6.4)	220 (19.9) 358 (32.4) 459 (41.5) 68 (6.2)
Depression categories						
Unlikely depression Likely depression Total	273 (40.2) 406 (59.8)	43 (42.2) 59 (57.8)	63 (52.5) 57 (47.5)	51 (40.8) 74 (59.2)	45 (57.7) 33 (42.3)	475 (43.0) 629 (57.0)
Anxiety categories						
Unlikely Anxiety Likely Anxiety Total	426 (63.1) 249 (36.9)	53 (52.5) 48 (47.5)	81 (68.1) 38 (31.9)	84 (67.2) 41 (32.8)	52 (67.5) 25 (32.5)	696 (63.4) 401 (36.6)
Prevalence of likely problems with mobility, self-care, usual activities, pain/discomfort, and anxiety/depression among respondents						
Dimension	Likely mobility problems	Likely self-care problems	Likely usual activities problems	Likely pain/discomfort	Likely depression/anxiety	
Prevalence (%)	11.8	8.2	65.8	77.7	89.2	

A chi-square test determined the association between demographic and clinical factors and the likelihood of two EQ-5D-5L dimensions: mobility and self-care. (**Table 2**).

**Table 2:** Chi-squared association test between the demographic and clinical antecedents and problems with mobility and self-care.

Variables	Likely mobility problem				Likely self-care problem			
	No issue N (%)	Issue N (%)	Chi-square	P-value	No issues N (%)	Issues N (%)	Chi-square	P-value
Demographic variables								
Age categories (years)								
≤25 years 26–40 years **>**40 years	318 (79.3) 298 (79.0) 209 (65.5)	83 (20.7) 79 (21.0) 110 (34.5)	22.7	.001	319 (77.3) 307 (81.4) 239 (74.9)	91 (22.70 70 (18.6) 80 (25.1)	4.46	.107
Gender								
Male Female Other Gender	350 (74.9) 455 (76.0) 20 (64.5)	117 (25.1) 144 (24.0) 11 (35.5)	2.09	.350	389 (83.3) 445 (74.3) 22 (71.0)	78 (16.7) 154 (25.7)	13.34	<.001
Ethnicity categories								
Caucasian Indigenous Black Asian Other	514 (76.1) 68 (67.3) 91 (76.5) 98 (78.4) 54 (70.1)	161 (23.9) 33 (32.7) 28 (23.5) 27 (21.6) 23 (29.9)	5.53	.237	521 (77.2) 73 (72.3) 103 (86.6) 97 (77.6) 62 (80.5)	154 (22.8) 28 (27.7) 16 (13.4) 28 (22.4) 15 (19.5)	7.56	.109
Education categories								
Less than High School High School Diploma Post-Secondary Educ Other	30 (73.2) 421 (74.6) 357 (77.8) 17 (51.5)	11 (26.8) 143 (25.4) 102 (22.2) 16 (48.5)	11.74	.008	31 (75.6) 439 (77.8) 370 (80.6) 16 (48.5)	10 (24.4) 125 (22.2) 89 (19.4) 17 (51.5)	18.74	<.001
Current relationship status								
Single Separated/Divorced Partnered/Married/Common law. Widowed Other	495 (76.5) 65 (75.6) 233 (72.8) 8 (66.7) 24 (75.0)	152 (23.5) 21 (24.4) 87 (27.2) 4 (33.3) 8 (25.0)	2.05	.727	506 (78.2) 69 (80.2) 249 (77.8) 7 (58.3) 25 (78.1)	141 (21.8) 17 (19.8) 71 (22.2) 5 (41.7) 7 (21.9)	2.98	.561
Current employment status								
Student Employed Unemployed Retired Other	76 (86.4) 266 (81.8) 419 (72.1) 33 (49.3) 31 (86.1)	12 (13.6) 59 (18.2) 162 (27.9) 34 (50.7) 5 (13.9)	43.03	.001	72 (81.8) 259 (79.7) 451 (77.6) 42 (62.7) 32 (88.9)	16 (18.2) 66 (20.3) 130 (22.4) 25 (37.3) 4 (11.1)	12.99	.001
Current housing status								
Own Home Rented Accommodation Live with Family/Friends Couch/Shelter/Street/Other	154 (70.3) 265 (75.1) 356 (77.9) 50 (73.5)	65 (29.7) 88 (24.9) 101 (22.1) 18 (26.5)	4.68	.196	178 (81.3) 272 (77.1) 358 (78.3) 48 (70.60	41 (18.7) 81 (22.9) 99 (21.7) 20 (29.4)	3.76	.288
Clinical variables								
PHQ categories								
Unlikely depression Likely depression	389 (82.4) 436 (69.8)	83 (17.6) 189 (30.2)	23.09	.001	422 (89.4) 434 (69.4)	50 (10.6) 191 (30.6)	62.53	<.001
GAD categories								
Unlikely anxiety Likely anxiety	550 (79.0) 275 (68.6)	146 (21.0) 126 (31.4)	14.88	.001	588 (84.5) 268 (66.8)	108 (15.5) 133 (33.2)	46.23	<.001

With regards to likely mobility problems, five variables namely age category (χ^2^ (2) = 22.7; p<0.001), education category (χ^2^ (3) = 11.7; p<0.008), current employment status (χ^2^ (4) = 43.0; p<0.001), PHQ9 category (χ^2^ (1) = 23.09; p<0.001), and GAD category (χ^2^ (1) = 14.88; p<0.001) showed significant association.

Also, for likely self-care problems, five variables namely gender category (χ^2^ (2) = 13.34; p<0.001), education category (χ^2^ (3) = 18.74; p<0.001), current employment status (χ^2^ (4) = 12.99; p<0.001), PHQ9 category (χ^2^ (1) = 62.53; p<0.001) and GAD category (χ^2^ (1) = 46.23; p<0.001) showed significant association.

**Table 3** illustrates the association between the demographic and clinical antecedents and the likelihood of three EQ-5D-5L domains, namely, the daily usual activity, pain/discomfort and depression/anxiety.

**Table 3:** Chi-squared association test between the demographic and clinical antecedents and likely problems with daily usual activity, pain/discomfort and depression/anxiety.

Variables	Likely daily activity problem				Likely pain/discomfort				Likely depression/anxiety			
	No issue N (%)	Issue N (%)	Chi-square	P-value	No issues N (%)	Issues N (%)	Chi-square	P-value	No issues N (%)	Issues N (%)	Chi-square	P-value
Demographic variables												
Age categories (years)												
≤25 years 26–40 years **>**40 years	213(53.1) 208 (55.2) 149 (46.7)	188 (46.9) 169 (44.8) 170 (53.3)	5.30	.071	169 (42.1) 173 (45.9) 122 (38.2)	232 (57.9) 204 (54.1) 197 (61.8)	4.13	.126	75 (18.7) 108 (28.6) 89 (27.9)	326 (81.3) 269 (71.4) 230 (72.1)	12.63	.002
Gender												
Male Female Other Gender	262 (56.1) 301 (50.3) 7 (22.6)	205 (43.9) 298 (49.7) 24 (77.4)	14.63	<.001	216 (46.0) 241 (40.2) 8 (25.8)	252 (54.0) 358 (59.8) 23 (74.2)	7.17	.028	151 (32.3) 118 (19.7) 3 (9.7)	316 (67.7) 481 (80.3) 28 (90.3)	26.37	<.001
Ethnicity categories												
Caucasian Indigenous People Black People Asians Other	328 (48.6) 54 (53.5) 77 (64.7) 66 (52.8) 45 (58.4)	347 (51.4) 47 (46.5) 42 (35.3) 59(47.2) 32(41.6)	12.23	.016	256 (37.9) 42 (41.6) 69 (58.0) 58 (46.4) 39 (50.6)	419 (62.1) 59 (58.4) 50 (42.0) 67 (53.6) 38 (49.4)	20.36	<.001	140 (20.7) 25 (24.8) 49 (41.2) 35 (28.0) 23 (29.9)	535 (79.3) 76 (75.2) 70 (58.8) 90 (72.0) 54 (70.1)	24.82	<.001
Education categories												
Less than High School High School Diploma Post-Secondary Educ Other	18 (43.9) 311 (55.1) 226 (49.2) 15 (45.5)	23 (56.1) 253 (44.9) 233 (50.8) 18 (54.5)	5.27	.153	18 (43.9) 242 (42.9) 198 (43.1) 6 (18.2)	23 (56.1) 322 (57.1) 261(56.9) 27 (81.8)	8.12	.043	10 (24.4) 136 (24.1) 122 (26.6) 4 (12.1)	31 (75.6) 428 (75.9) 337 (73.4) 29 (87.9)	3.77	.287
Current relationship status												
Single Separated/Divorced Partnered/Married/Common law. Widowed Other	354 (54.7) 35 (40.7) 156 (48.8) 5 (41.7) 20 (62.5)	293 (45.3) 51 (59.3) 163 (51.2) 7 (58.3) 12 (37.5)	9.60	.048	290 (44.8) 34 (39.5) 129 (40.3) 3 (25.0) 8 (25.0)	357 (55.2) 52 (60.5) 191 (59.7) 9 (75.0) 24 (75.0)	7.86	.096	171 (26.4) 19 (22.1) 75 (23.4) 3 (25.0) 4 (12.5)	476 (73.6) 67 (77.9) 245 (76.6) 9 (75.0) 28 (87.5)	4.17	.383
Current employment status												
Student Employed Unemployed Retired Other	49 (55.7) 157 (48.3) 310 (53.4) 28 (41.8) 26 (72.2)	39 (44.3) 168 (51.7) 271 (46.6) 39 (58.2) 10 (27.8)	11.37	.023	44 (50.0) 167 (51.4) 225 (38.7) 16 (23.9) 12 (33.3)	44 (50.0) 158 (48.6) 356 (61.3) 51 (76.1) 24 (66.7)	26.66	<.001	19 (21.6) 92 (28.3) 138 (23.8) 19 (28.4) 4 (11.1)	69 (78.4) 233 (71.7) 443 (76.2) 48 (71.6) 32 (88.9)	7.04	.134
Current housing status												
Own Home Rented Accommodation Live with Family/Friends Couch/Shelter/Street/Other	98 (44.7) 182 (51.6) 263 (57.5) 27 (39.7)	121 (55.3) 171 (48.8) 194 (42.5) 41 (60.3)	14.40	.002	91 (41.6) 142 (40.2) 210 (46.0) 21 (30.9)	128 (58.4) 211 (59.8) 247 (54.0) 47 (69.1)	6.80	.079	64 (29.2) 80 (22.7) 113 (24.7) 15 (22.1)	155 (70.8) 273 (77.3) 344 (75.3) 53 (77.9)	3.43	.329
PHQ categories												
Unlikely depression Likely depression	338 (71.6) 232 (37.1)	134 (28.4) 393 (62.9)	128.15	<.001	261 (55.3) 203 (32.5)	211 (44.7) 422 (67.5)	57.36	<.001	213 (45.1) 59 (9.4)	259 (54.9) 566 (90.6)	183.66	<.001
GAD categories												
Unlikely anxiety Likely anxiety	437 (62.8) 133 (33.2)	259 (37.2) 268 (66.8)	89.42	<.001	329 (47.3) 135 (33.7)	367 (52.7) 266 (66.3)	19.29	<.001	232 (33.3) 40 (10.0)	464 (66.7) 361 (90.0)	74.44	<.001

Concerning likely problems with daily usual activity, seven variables showed significance and one near significant association, including, age (χ^2^ (2) = 5.30; p<0.071), gender (χ^2^ (2) = 14.63; p<0.001), ethnicity category (χ^2^(4) = 12,23; p<0.016), relationship status (χ^2^ (4) = 9.60; p<0.048), current employment status (χ^2^(4) = 11.37; p<0.023), housing category χ^2^((3) = 14.40; p<0.002), PHQ9 category (χ^2^ (1) = 128.15; p<0.001) and GAD category (χ^2^ (1) = 89.42; p<0.001).

Additionally, six variables showed significance, and one near-significant association was found regarding likely pain and discomfort problems. They were gender category (χ^2^ (2) = 128.15; p<0.028), ethnicity category (χ^2^ (4) = 20.36; p<0.001), educational status (χ^2^ (3) = 8.12; p<0.043), employment status (χ^2^ (4) = 26.66; p<0.001), current housing status (χ^2^ (4) = 6.80; p<0.079, PHQ9 category (χ^2^ (1) = 57.36; p<0.001) and GAD category (χ^2^ (1) = 19.29; p<0.001).

Finally, five variables were identified to have a significant association with likely depression and anxiety. These were age category (χ^2^ (2) = 12.63; p<0.002), gender category (χ^2^ (2) = 26.37; p<0.001), ethnicity category (χ^2^ (4) = 24.32; p<0.001), PHQ9 category (χ^2^ (1) = 183.66; p<0.001) and GAD category (χ^2^ (1) = 76.44; p<0.001.

**Table 4**, as illustrated, used the binary logistic regression model to predict likely problems with mobility, self-care, usual activities, pain/discomfort, and depression/anxiety among respondents. The five models included various variables that showed a significant or near significant association, such as Age, p<.071, for the likely problems with the daily activities model, and current relationship status, p<0.096 and housing status, p<0.079, for the pain and discomfort model on Chi-Square analysis.

**Table 4:** Bivariate binary logistic regression model that predicts the likely issues with mobility, self-care, usual activities, pain/discomfort, and depression/anxiety.

Variables	Likely mobility Issues			Likely self-care issue			Likely daily activity issues			Likely pain/discomfort			Likely depression/anxiety		
	OR	*P*-value	95% CI	OR	*P*-value	95% CI	OR	*P*-value	95% CI	OR	*P*-value	95% CI	OR	*P*-value	95% CI
Demographic variables															
Age categories															
(years) ≤25 years 26–40 years **>**40 years	1.13 1.98	.002 .541 **<.**001	.769 -1.65 1.32 - 2.97				.837 1.06	.312 .796	.594 -1.18 .691 - 1.61				.543 .547	.003 .002 .003	.371-.796 .366-.819
Gender															
Male Female Other				1.60 1.57	.017 .005 .303	1.16- 2.22 .667 - 3.69	1.08 3.65	.025 .577 .007	.822- 1.42 1.44- 9.29	1.16 2.12	.167 .270 .088	.892 - 1.50 .894 - 5.03	1.66 2.13	.005 .001 .263	1.21- 2.27 .566- 8.03
Ethnicity categories															
Caucasian Indigenous People Black People Asians Other							.415 .040 .819 .618	.337 .823 .623 .951 .873	.514 -1.32 .396-.979 .620-1.46 .513- 1.49	.797 .467 .799 .745	.012 .331 <.001 .291 .251	.504 -1.26 .305-.717 .527- 1.21 .451- 1.23	.334 .566	<.001 <.001 .023	.207-.540 .346-.926
Education categories															
Less than High School High School Diploma Post-Secondary Educ Other	1.08 .723 2.66	.004 .825 .401 .061	.518- 2.28 .339- 1.54 .955- 7.41	.935 .640 4.04	**<**.001 .863 .267 .010	.432 - 2.02 .290 - 1.41 1.39-11.71				1.33 1.26 3.70	.139 .412 .508 .025	.675- 2.61 .633- 2.52 1.18- 11.59			
Current employment status															
Student Employed Unemployed Retired Other	1.21 2.12 5.05 .739	**<**.001 .601 .028 <.001 .616	.594 -2.46 1.09-4.14 2.1 - 12.04 .226 - 2.41	1.25 1.43 4.69 .396	**<**.001 .494 .247 **<.**001 .159	.660 - 2.36 .781 - 2.61 2.12-10.39 .109 - 1.44	1.23 1.19 1.78 .412	.480 .528 .154 .103	.695- 2.17 .698- 2.02 .806- 3.93 .142- 1.19	.808 1.55 3.18 1.25	**<.**001 .430 .081 .004 .663	.475 - 1.37 .947 - 2.53 1.45 - 6.96 .466 - 3.33			
Clinical variables															
PHQ categories															
Unlikely depression	1.89	**<**.001	1.34 - 2.66	2.87	**<**.001	1.94 - 4.23	3.22	**<**.001	2.39 - 4.34	2.31	**<**.001	1.72 - 3.10	5.72	<.001	3.97 - 8.25
GAD categories															
Unlikely anxiety	1.63	.004	1.16 - 2.27	1.99	**<**.001	1.41- 2.81	2.03	**<**.001	1.49 - 2.75	1.28	.124	.935 - 1.74	1.82	.006	1.19 - 2.79

### 3.1 Regression models

#### 3.1.1 Mobility model

Five variables, namely age category, education category, current employment status, PHQ-9 category, and GAD category, showed a significant association and were included in the regression model. The model was statistically significant: χ^2^ (df=11; n=1106) = 75.20, p<.001 for likely problem with mobility among study respondents, indicating that the model could differentiate between respondents who did or did not exhibit a likely problem with mobility. The model accounted for 8.8% (Cox and Snell R2) to 13.0% (Nagelkerke R2) of the variance and accurately classified as 76.2% for likely problems with mobility among study respondents. It can be observed that five main variables, namely, age, ethnicity, educational status, employment status, likely depression, and likely anxiety, could significantly predict the likely problems with mobility among respondents, as illustrated in **Table 4**.

Concerning likely mobility problems, it can be observed that participants who were above 40 years old were almost two times more likely to present with mobility problems (OR=1.98; 95% C.I. = 1.32 - 2.97) compared to those who were 25 years old or less. Also, participants in the other category of educational status were a little over two and a half times more likely to present with mobility problems (OR=2.66; 95% C.I.955 - 7.41) compared with those with high school diplomas. Furthermore, unemployed participants were two times (OR=2.12; 95% C.I. = 1.09 - 4.14), and participants who were retired were five times more likely to present with mobility problems (OR=5.05; 95% C.I. = 2.10 - 12.04), respectively, compared to those who were employed. Additionally, participants who had likely depression were almost two times more likely to present with mobility problems (OR=1.89; 95% C.I. = 1.34 - 2.66) compared to participants with unlikely depression. Finally, participants with likely anxiety were a little over one and a half times more likely to present with mobility problems (OR=1.63; 95% C.I. = 1.16 - 2.27) compared with participants who had unlikely anxiety.

#### 3.1.2 Self-care model

Also, five variables, namely, gender category, education category, current employment status, PHQ9 category, and GAD category, showed a significant association and were included in the model. The model was statistically significant; χ^2^ (df=21; n=1106) = 52.00, p<.001 for self-care among study respondents, indicating that the models could differentiate between respondents who did or did not exhibit likely self-care problems. The model accounted for 11.0% (Cox and Snell R2) to 16.9% (Nagelkerke R2) of the variance and accurately classified as 78.8% for likely problems with self-care among study respondents. It can be observed that five main variables, namely, age, ethnicity, educational status, employment status, likely depression, and likely anxiety, could significantly predict the likely problems with self-care among respondents, as illustrated in **Table 4**. Regarding likely self-care-related problems, participants identified as females were slightly over one and a half times more likely to present with self-care problems (OR=1.60; 95% C.I. = 1.16 - 2.22) than their male counterparts. Also, participants in the Other category of educational status were four times more likely to present with self-care problems (OR=4.04; 95% C.I. = 1.39 - 11.71) than those with high school diplomas. Retired participants were four and a half times more likely to present with self-care problems (OR=4.69; 95% C.I. = 2.12 - 10.39) than those employed. Also, participants who had likely depression were almost three times more likely to present with self-care problems (OR=2.89; 95% C.I. = 1.94 - 423) compared to participants with unlikely depression. Finally, participants with likely anxiety were almost two times more likely to present problems relating to self-care (OR=1.99; 95% C.I. = 1.41 - 2.81) compared with those who had unlikely anxiety.

#### 3.1.3 Daily usual activity model

Furthermore, seven variables showed significance, and one near-significant association, namely, age, gender, ethnicity category, relationship status, current employment status, housing category, PHQ9 category, and GAD category, were included in the regression model. The model was statistically significant; χ^2^ (df=19; n=1106) = 52.00, p<.001 for daily usual activity among study respondents, indicating that the models could differentiate between respondents who did or did not exhibit likely problems with usual activities. The model accounted for 10.3% (Cox and Snell R2) to 13.8% (Nagelkerke R2) of the variance and accurately classified as 64.6% for likely problems with usual activities among study respondents. It can be observed that three main variables, namely, gender, likely depression, and likely anxiety, could significantly predict the likely problems with daily usual activity among respondents, as illustrated in **Table 4**.

Regarding likely problems relating to daily activities, participants who identified as “Other” gender were three and a half times more likely to present problems regarding usual daily activities (OR=3.22; 95% C.I. = 1.44 - 9.29) compared to the male gender. Also, participants who had likely depression were three times more likely to present with problems with usual daily activities (OR=3.22; 95% C.I. = 2.29 - 4.34) compared to participants with unlikely depression. Finally, in terms of presenting problems of usual daily activities, participants with likely anxiety were two times more likely to show these problems (OR=2.03; 95% C.I. = 1.49 - 2.75) compared with those with unlikely anxiety.

#### 3.1.4 Pain/discomfort model

With pain and discomfort problems, six variables showed significance and one near-significant association and were included in the regression model. They were categorized by gender, ethnicity, educational status, employment status, current housing status, PHQ-9 category, and GAD category. This model was also statistically significant: χ^2^ (df=22; n=1106) = 57.70, p<.001 for likely pain/discomfort among study respondents, indicating that the models could differentiate between respondents who did or did not exhibit likely problems with pain and discomfort. The model accounted for 10.7% (Cox and Snell R2) to 14.4% (Nagelkerke R2) of the variance and accurately classified as 64.5% for likely problems with pain/discomfort among study respondents. Three main variables, namely, ethnicity, employment status, and likely depression, could significantly predict the likely problems with pain/discomfort among study respondents, as illustrated in **Table 4**.

In looking at likely pain/discomfort, it can be observed that Caucasians were two times more likely to present with problems regarding pain and discomfort (OR=2.14; 95% C.I. = 1.39 - 3.27) compared to Black persons. Also, retired participants were three times more likely to present with pain/discomfort (OR 3.18; 95% C.I. = 1.45 - 6.96) than those employed. Finally, participants with likely depression were two times more likely to present with pain/discomfort (OR=2.31; 95% C.I. = 1.72 - 3.10) compared to those with unlikely depression.

#### 3.1.5 Depression/anxiety model

Finally, five variables were identified to have a significant association with likely depression/anxiety and were included in the regression model. These were age categories, gender category, ethnicity category, PHQ-9 scale-based Depression category, and GAD-7 scale-based Anxiety category. The model was statistically significant; χ^2^ (df=10; n=1106) = 75.2, p<.001 for depression/anxiety among study respondents, indicating that the models could differentiate between respondents who did or did not exhibit likely problems with anxiety and depression. The model accounted for 19.5% (Cox and Snell R2) to 29.0% (Nagelkerke R2) of the variance and accurately classified as 78.6%. Respectively, for likely problems with depression/anxiety among study respondents. It can be observed that five main variables, namely, age, gender, ethnicity, likely depression, and likely anxiety, could significantly predict the likely problems with depression/anxiety among study respondents, as illustrated in **Table 4**.

Finally, when it comes to problems relating to likely depression/anxiety, participants above 40 years old were also almost two times more likely to present with depression/anxiety problems (OR=1.82; 95% C.I. = 1.22 - 2.73) compared to those less than 25 years old. Also, participants who identified as females were a little over one and a half times more likely to present with depression/anxiety (OR=1.66;95% C.I. = 1.21 - 2.27) compared to their male counterparts. Furthermore, Caucasians were almost three times (OR=2.99; 95% C.I. = 1.85 - 4.83) and one and a half times (OR=1.76; 95% C.I. = 1.07 - 2.89) more likely to present with depression/anxiety problems compared to Black persons and participants who identify as Asians, respectively. Also, participants with likely depression were a little over five and a half times more likely to present with depression/anxiety problems (OR=5.72; 95% C.I. = 3.97 - 8.25) compared to those with unlikely depression. Finally, in terms of presenting problems with depression/anxiety, participants who had likely anxiety were almost two times (OR=1.82; 95% C.I. = 1.19 - 2.79) more likely compared to participants with unlikely anxiety.

## 4 Discussion

The main objective of this paper was to explore the predictors of poor Qol outcomes across the five dimensions of the EQ-5D-5L, namely mobility, self-care, usual activities, pain/discomfort and depression/anxiety for individuals discharged from acute psychiatric care in Alberta. The study’s outcome identified multiple variables, such as age, gender, employment status, educational background, and mental health conditions, as patterns and risk factors that significantly impact participants’ health-related challenges. This discussion will explore the key findings from the regression outputs and interpret their potential implications for individual health management and broader health interventions.

### 4.1 Mobility problems

The prevalence of mobility problems is 11.8%. This is lower than 60.1% reported in a study that sought to assess the health-related quality of life (HRQoL) and identify its predictors among type 2 diabetes patients of Bangladesh (52). The analysis of mobility problems reveals that certain demographic factors significantly increase the likelihood of experiencing these challenges. Participants aged 26 to 40 were almost twice as likely to present with mobility problems compared to younger individuals aged 25 years or less. This finding is consistent with literature suggesting that the early stages of adulthood may be associated with an increasing risk of musculoskeletal disorders or chronic conditions that affect mobility (53–55). According to reports, around 1.71 billion people globally suffer from musculoskeletal conditions. These conditions are the primary cause of disability worldwide, with low back pain being the leading cause of disability (56). Musculoskeletal disorders severely restrict mobility and dexterity, often leading to early retirement, lower well-being, and a diminished capacity to engage in social activities (56, 57).

The transition into middle age, typically involving lifestyle changes such as weight gain or decreased physical activity, could further exacerbate mobility concerns in this age group (58–60).

The finding that participants with likely depression were almost twice as likely to experience mobility problems highlights the significant interplay between mental and physical health. Depression can manifest in physical symptoms such as fatigue, pain, and reduced physical activity, all of which could contribute to mobility limitations (61, 62). Similarly, participants with likely anxiety were one and a half times more likely to report mobility problems. Anxiety can result in physical tension, muscle stiffness, and hypervigilance, leading to movement difficulties (63, 64). Thus, mental health conditions should be closely monitored and treated alongside physical health concerns to reduce mobility impairments (65).

Additionally, retired individuals were five times more likely to experience mobility problems. Retirement, while often a time for rest, can also bring about deteriorated physical health due to decreased physical activity (66). Those who are unemployed were also at an increased risk for mobility challenges, indicating that limited access to healthcare or a more sedentary lifestyle associated with unemployment may contribute to physical decline (67, 68). These findings suggest that retirement and unemployment should be critical for targeted health interventions to maintain mobility.

### 4.2 Self-care problems

The prevalence of self-care problems is 8.3%. Self-care challenges are another significant health problem, and this study identifies several factors that increase the risk of such difficulties. Female participants were more likely to experience self-care issues, which is in line with prior research suggesting that women may be more prone to conditions that require more intensive self-care due to factors like hormonal changes, caregiving roles, and higher rates of certain chronic conditions, such as arthritis and fibromyalgia (69). These finding calls attention to the need for gender-sensitive healthcare approaches that consider women’s unique self-care challenges.

Moreover, individuals in the “Other” category of educational status were four times more likely to experience self-care problems. The above result suggests a strong association between lower educational attainment and difficulties with self-care. Lower level or no forms of education may be linked to reduced health literacy, poorer access to healthcare resources, and less knowledge about maintaining health, all of which contribute to challenges with self-care. Interventions focusing on health education and empowerment could significantly benefit these groups (70, 71).

Retired participants were more than four times more likely to face self-care challenges. The result is consistent with the notion that aging and physical decline accompanying retirement can lead to difficulty maintaining personal care routines (59, 72). Retirees may face increased isolation, decreased mobility, and physical conditions like arthritis, which contribute to self-care problems. Targeted programs that promote physical activity, mental health support, and accessible healthcare services could help mitigate these challenges for retirees.

Finally, the strong relationship between likely depression and self-care problems emphasizes the need to integrate mental health care with support for physical well-being. Those experiencing depression may lack the motivation or energy to engage in proper self-care, further exacerbating their health conditions (73).

### 4.3 Daily activity problems

The prevalence of daily activity problems is 65.8%. The high levels of daily activity issues harm social and occupational functioning. Difficulty with self-care can worsen stress levels, leading to depression, anxiety and other mental health problems. When examining the challenges related to daily activities, several demographic and mental health factors emerged as significant contributors. Participants who identified as “Other” gender were three and a half times more likely to experience problems with daily activities. This finding suggests that individuals who do not fit into traditional gender categories may face unique social or health-related challenges, possibly stemming from societal marginalization, lack of appropriate healthcare, or difficulties in accessing support networks (74, 75). These findings emphasize the importance of inclusive healthcare systems that recognize and address the needs of non-binary individuals.

Depression was also found to be a strong predictor of difficulties with daily activities, with those who are likely to be depressed three times more likely to face these challenges. Depression can significantly impair cognitive function and energy levels, making it difficult to complete routine tasks (76). Furthermore, the presence of likely anxiety increased the likelihood of daily activity problems. Anxiety can lead to difficulties in concentration and decision-making, and physical restlessness, which all interfere with daily functioning (77).

### 4.4 Pain/discomfort problems

The prevalence of pain/discomfort problems is 77.7%. This is comparable to 77.7% found in a study that sought to assess the health-related quality of life (HRQoL) and identify its predictors among type 2 diabetes patients of Bangladesh (52). The high levels of pain/discomfort can increase the potential for reduced mobility and self-care, reduce the quality of life, and negatively impact social and occupational functioning (78, 79). Pain and discomfort are prevalent problems that impact individuals’ quality of life, and the regression analysis reveals several critical factors influencing these symptoms. Caucasian participants were found to be twice as likely to experience pain/discomfort compared to Black participants. The results suggest that racial disparities may exist in the experience or reporting of pain (80, 81). There may be cultural, socioeconomic, or healthcare access factors at play that lead to differential reporting or treatment of pain across racial groups (82). It is essential to explore these factors further to address healthcare disparities effectively.

Retirement also emerged as a significant factor, with retired individuals being three times more likely to experience pain/discomfort. The combination of aging and the reduced physical activity associated with retirement can contribute to chronic pain conditions such as arthritis or muscle stiffness (83, 84). The results underscore the importance of creating programs promoting physical activity and pain management for older adults, particularly those who are retired.

Finally, depression was strongly associated with pain/discomfort. The relationship between mental health and physical pain is well-established, as depression can exacerbate the perception of pain and contribute to chronic pain conditions (85–87). This finding reinforces the need for integrated care that addresses mental and physical health to improve the overall well-being of individuals experiencing pain/discomfort (88, 89).

### 4.5 Depression/anxiety problems

The prevalence of depression/anxiety is 89.2%. This is higher than the 81.1% reported and in a study explored the ability of the EQ-5D Health Related Quality of Life measure to differentiate among those with a clinical diagnosis of major depressive episodes and/or anxiety disorders among respondents (90). These high levels of depression/anxiety increase the risks of mental and physical health conditions, employment and academic challenges, and substance misuse (91–93). It can reduce the quality of life and increase health service utilization (24, 45). Depression and anxiety are both highly influential factors in the overall health outcomes observed in this study. Depression having a profound effect on quality of life (Qol), disrupting multiple areas of daily functioning, such as physical health, social relationships, and general well-being. There is a relationship between lower Qol and both psychological and somatic symptoms of depression, with somatic symptoms often serving as stronger predictors of reduced quality of life (16, 94). Participants over 40 years old were nearly twice as likely to report depression and anxiety problems compared to younger individuals. This finding may reflect the cumulative effect of aging on both physical and mental health. As individuals age, they may experience more significant physical health challenges, loss of loved ones, or increased caregiving responsibilities, all of which could heighten the risk of depression and anxiety (95–97).

Female participants were more likely to present with depression/anxiety, further highlighting the gendered nature of mental health concerns. The greater emotional and social pressures on women, as well as potential gender disparities in healthcare access or treatment, could contribute to higher rates of depression and anxiety in this group (98, 99).

Caucasian participants were found to be nearly three times more likely to experience depression/anxiety compared to Black participants. The outcome above could point to disparities in how depression and anxiety are experienced or managed across racial groups. Cultural factors, healthcare access, and social determinants of health may all play a role in these differences (100–102).

Finally, the presence of likely depression and likely anxiety dramatically increased the likelihood of presenting with both depression/anxiety problems (103, 104). The finding emphasizes the interconnectedness of mental health conditions and the need for comprehensive mental health interventions that address both conditions simultaneously (105).

### 4.6 Implications for policy and practice

The findings of this study offer critical insights into the multifaceted determinants of poor quality of life (Qol) outcomes among individuals recently discharged from acute psychiatric care in Alberta. These insights have profound implications for policy development and clinical practice. The predictors identified across the EQ-5D-5L dimensions—mobility, self-care, usual activities, pain/discomfort, and anxiety/depression—underscore the necessity of adopting an integrated, person-centered approach to mental and physical health interventions.

From a policy perspective, the strong association between mental health conditions, particularly depression and anxiety, and physical impairments such as mobility and self-care difficulties highlight the urgent need for integrated care models. These models should prioritize collaborative care frameworks that bring together mental health professionals, primary care providers, and rehabilitation services to address both psychological and somatic symptoms in tandem. Government funding and health authority support should be directed toward multidisciplinary outpatient programs explicitly designed for psychiatric discharge populations, emphasizing early intervention and continuity of care.

The data also emphasize the importance of addressing social determinants of health, including employment status, education, and retirement. The elevated risk of mobility and self-care challenges among retired and unemployed individuals calls for targeted policy measures that promote physical activity, social engagement, and accessible community support for these groups. Health promotion programs tailored to older adults and those not in the labor force, especially those living alone or with limited mobility, should be scaled and adapted to reduce isolation and maintain functional independence.

Educational disparities also emerged as significant, particularly among individuals categorized under “Other” educational backgrounds, who were more prone to self-care and daily activity limitations. This finding advocates for policies that enhance health literacy, particularly among marginalized or undereducated populations. Health education initiatives, delivered through community health centers, adult learning programs, and culturally tailored outreach, can empower individuals with the knowledge and resources to manage their physical and mental health more effectively.

Gender and racial disparities observed in this study further indicate the need for equity-informed health policies. Women were more likely to experience self-care and mental health issues, likely due to a complex interplay of caregiving roles, hormonal factors, and societal expectations. Policy responses must support gender-sensitive healthcare services, including access to mental health support, caregiver respite programs, and routine screening for chronic conditions disproportionately affecting women.

The greater likelihood of pain/discomfort and anxiety/depression among Caucasian participants compared to Black participants suggests the presence of culturally mediated differences in healthcare access, symptom reporting, or coping strategies. These findings support the development of culturally competent healthcare services that consider how race, identity, and lived experiences shape mental and physical health outcomes. Enhanced training for healthcare providers in cultural safety and anti-racism practices is essential to reducing disparities and building trust among diverse patient populations.

Finally, for clinical practice, the consistent role of depression and anxiety across all Qol domains supports routine mental health screening post-discharge and the inclusion of psychological support in all care plans. Clinicians should receive training to recognize the physical manifestations of mental illness and vice versa, ensuring holistic assessments and interventions. Together, these measures can significantly improve post-discharge outcomes and long-term recovery for individuals transitioning from psychiatric care back into the community.

## 5 Limitations

One limitation of this study is the absence of a well-defined control group, which reduces the ability to generalize the results effectively. Additionally, selection bias is a concern, as participants were required to have an active cell phone to receive the proposed intervention. Almost 100 participants were excluded from the study for the reason of not having a mobile phone. As a result, we could not capture individuals without active cell phones’ clinical characteristics and quality of life measures. Another limitation is that, while gender data was collected, we did not gather information on participants’ biological sex. Consequently, we could not investigate biological sex as a potential influencing factor, nor did we account for individuals whose sex at birth differed from their identified gender. Finally, the study did not include predictors such as diagnostic variables and medical co-morbidities which are known to impact quality of life. Despite these limitations, this large-scale study offers valuable insights into the prevalence and predictors of low Qol among patients with mental health challenges admitted to acute care facilities in Alberta.

## 6 Conclusion

The results of this study highlight the complex interplay of demographic, socioeconomic, and mental health factors that influence various health-related problems. Factors such as age, gender, employment status, educational background, and mental health conditions significantly increase the likelihood of experiencing mobility, self-care, daily activity, pain/discomfort, depression/anxiety problems. These findings underscore the importance of targeted, holistic health interventions that address physical and mental health needs. Programs and policies that consider these risk factors will be more effective in improving the overall health outcomes of diverse populations. Moreover, a focus on mental health, particularly depression and anxiety, is crucial, as these conditions are consistently linked to a range of physical and functional health challenges.

## Data Availability

The raw data supporting the conclusions of this article will be made available by the authors, without undue reservation.
